# Work-related stress, quality of life, and coping mechanism among lecturers in a Tertiary Educational Institution in Anambra State, Nigeria

**DOI:** 10.1186/s40359-023-01114-5

**Published:** 2023-03-16

**Authors:** Uchechukwu Martha Chukwuemeka, Uchenna Prosper Okonkwo, Chibuike Jefferen Njoku, Sylvester Emeka Igwe, Taiwo Joseph Oyewumi, Daniel Chimmuanya Ugwuanyi

**Affiliations:** 1 Department of Medical Rehabilitation, Faculty of Health Sciences and Technology, Nnamdi Azikiwe University, Awka, Nigeria; 2 Department of Medical Rehabilitation, University of Nigeria, Nsukka, Nigeria; 3 Department of Physiotherapy, University of Benin Teaching Hospital, Edo State Benin City, Nigeria; 4 Department of Radiography and Radiological Sciences, Faculty of Health Sciences and Technology, Nnamdi Azikiwe University, Awka, Nigeria

**Keywords:** Coping mechanism, Lecturers, Quality of life, Nigeria, Tertiary Institution, Work-related stress

## Abstract

**Introduction:**

Work-related stress (WRS) is a highly prevalent and pervasive problem that can result in loss of productivity and deterioration of a lecturer’s health. Lecturing work requires coping with some of the stressful situations found in any workplace to have a favourable quality of work life. The study determined the influence of sex, years of teaching experience, and academic rank on work-related stress, coping mechanisms, and quality of work life among lecturers at Nnamdi Azikiwe University (NAU).

**Method:**

This was a cross-sectional survey involving 283 lecturers consecutively recruited from NAU after proportionate randomization of the lecturers in 101 departments. The Health and Safety Executive Work Related stress (HSE-WRS), Work-Related Quality of life (WRQL), and Brief-cope Questionnaires (BCQ) were applied to assess the participant’s work-related stress, quality of work life, and coping mechanism (CM) respectively. Data were analyzed using Kruskal Wallis and Mann-Whitney U tests at a 0.05 level of significance.

**Result:**

Sex, years of teaching experience, and academic rank had statistically significant influence on 14 subsets of coping mechanism with p-values </=0.01. Years of teaching experience had a statistically significant influence on work-related stress (p = 0.00). Sex, years of teaching experience, and academic rank did not influence work-related quality of life in a statistically significant way.

**Conclusion:**

There was a statistically significant influence of sex, years of teaching experience, and academic rank on coping strategies of lecturers. Also, a statistically significant influence of years of teaching experience on work related stress of lecturers was ascertained and revealed that male lecturers coped better with the rigorous demands of the job compared to female lecturers.

## Introduction

Stress was conceived as pressure from the environment, then as strain within the person [1]. Stress is generally a situation where the demands exceed the capacity of an individual to respond and can potentially have negative physical and psychological consequences [2, 3]. Thus, stress is more likely in some situations than others and in some individuals than others. Stress can undermine the achievement of goals, both for individuals and for organizations [1]. Stress was also defined as an adaptive response, mediated by individual differences and/or psychological processes that are a consequence of any external (environmental) action, situation, or event that places excessive psychological and/or physical demands on a person [4]. A stressor is an environmental event that significantly perturbs the entire human dynamical system away from the optimal attractor resulting in a state of lower utility [5]. Stressors are not necessarily physical changes in the environment but may involve the loss of a significant relationship, financial stress, negative neighborhood characteristics, or social threats including discrimination [6–9]. Work-related stress has been defined as harmful physical and emotional responses that occur when the requirements of the job do not match the capabilities, resources, or needs of the worker [10]. Work-related stress has become a major occupational risk factor in all industrialized countries, although comparatively less is known within many newly industrialized and developing countries [11]. The experience of workplace stress has been subject to a large amount of research and interest in the topic shows no sign of waning. It is now generally accepted that prolonged intense stress can hurt an individual’s mental and physical health [12, 13], and coping mechanism to relief stress are often times adopted.

Coping mechanisms were defined as constantly changing cognitive and behavioral efforts to manage specific external and/or internal demands that are appraised as taxing or exceeding the resources of the person [14]. Coping is dependent on personality and perceptions about life experiences and the strategies adopted can differ by individuals. However, overall, the main aim is to reduce stress, reach a balanced state of functioning [15] and quality of life. Quality of work life (QOWL) is the degree to which members of a work organization can satisfy important personal needs through their experiences in the organization [16]. Quality of work life is a systemic approach that affects all aspects of the organization and forms a series of beliefs and values [17, 18]. The most distinct element of QOWL is its relationship to the impact not only on the employers but also on organizational efficiency and organizational decision-making processes. In general, quality of work life is an employee’s visible physical and mental vitality and the level of job satisfaction and motivation. Quality of work life is the provision by employers of a working environment that supports employees’ efficiency, productivity, morale, and motivation by identifying the preferred or non-preferred elements [17, 18].

It has been noted that a lack of awareness and research in the area of psychosocial risks and work-related stress hampers action in developing countries [11]. The authors were concerned that the proliferation of universities (public and private universities) in Nigeria and the corresponding increase in the enrolment of students who want to have undergraduate training in various areas of interest might impact work-related stress, quality of life, and coping mechanisms of the lecturers. It is anticipated that for the lecturers to deliver their utmost best to the students they need to have reduced work-related stress, improved quality of life, and good coping strategies. We have through a literature search discovered that there is a dearth of studies on the levels of work-related stress, quality of life, and coping mechanisms and how sex, years of teaching experience, and academic rank influences these variables among lecturers. This has created a knowledge gap in this area of study and has necessitated the need for the researchers to undertake the current study. The anticipation is that the outcome would highlight the need for the respective authorities to take necessary measures to improve the working environment of the lecturers to be more conducive by putting the necessary stress-reducing measures in place to mitigate work-related stress and promote work related quality of life.

## Methods

### Research design

A cross-sectional survey among 342 participants was the study design. Taro Yamane formula [19] was used to calculate the sample size as follows: N/1 + N(e)² = 2511/1 + 2511(0.05)² = 345; where population size (N) of the lecture is 2,511 and e is margin of error. A proportionate stratified random sampling technique was used to select respondents that participated in the study at department level and individual participants were consecutively sampled. The proportions of lectures from 101 departments (from 14 faculties) from 4 campuses of one university were calculated, each department sample contains the same sampling fraction of the population size. A total of 400 respondents were given the paper-based questionnaires, 342 returned the questionnaire, and 59 out of these were not utilized because of incomplete questionnaires; response rate was 85.5% .

### Research instrument

#### Socio-demographic form

A general information form used to collect participants’ age, marital status, sex, faculty, academic rank, and years of teaching experience.

#### Health and safety work-related stress questionnaire

The Health and Safety Executive (HSE), based in the United Kingdom, proposed this 35-item indicator tool for tracking work-related stress. It relates to the six stressors listed in the management standards approach to combating WRS. Organizations are using it more frequently to keep an eye on circumstances that can cause stress. It has > 0.75 validity and reliability rating in Cronbach’s alpha [20]. Manual scoring is employed. The score for the 35 questions from the HSE WRS Questionnaire, commonly known as an indicator tool, provides feedback on one’s performance in relation to HSE standards. The Likert scale has five levels for each item. Scores for each item range from 1 (poor) to 5 (desirable). Calculating the average and total scores for each participant was done in order to score each participant.

#### The coping mechanism questionnaire (brief cope)

A 28-item self-report questionnaire called the Coping mechanism questionnaire (Brief-COPE) was created to assess the effectiveness of coping strategies for stressful life events. The 60-item COPE scale, which was theoretically constructed based on several models of coping, was reduced to the Brief-Cope and was initially validated on a community sample of 168 hurricane victims [21]. A wide definition of “coping” is an effort to lessen the suffering from unpleasant life experiences. On a scale from 1 (I haven’t been doing this at all) to 4 (I have been doing this a lot), participants rate the frequency of usage of 28 coping behaviors and ideas (two items for each subscale). The coping strategy subscales include self-distraction, denial, substance abuse, behavioral disengagement, emotional support, venting, humor, acceptance, self-blame, religion, active coping, the use of instrumental support, positive reframing, and planning. The 14 subscales’ internal reliability values range from 0.57 to 0.90. The total scores on each subscale vary from two (the lowest) to eight (maximum); Summing the proper elements for each subscale results in better scores. No items are scored in reverse. There is simply a total score for each of the subscales; there is no overall total score [22].

#### Work-related quality of life questionnaire

The Work-Related Quality of Life (WRQoL) scale is a 23-item psychometric scale used to gauge the perceived quality of life of employees as measured through six psychosocial sub-factors and an overall score [23]; The latter is what we utilized in this study. The Likert format was scored thus: “Strongly Agree” = 5, “Agree” = 4, “Neutral” = 3, “Disagree” = 2, and “Strongly Disagree” = 1, according to the data’s coding. Higher scores thus denote greater agreement. The three negatively phrased items’ scores are inverted. After coding, higher scores indicate a greater perceived quality of working life. The overall WRQoL factor score is determined by finding the average of all 23 WRQoL items. A validation and reliability exercise conducted with the WRQoL scale in 2008 and 2009, [24] reported the overall Cronbach’s alpha for all 23 items to be an excellent 0.94.

### Procedure for data collection

Ethical approval was sought and obtained from the Faculty of Health Ethical Review Committee of Nnamdi Azikiwe University, Nnewi campus. An informed consent form that stated the study objectives, the data collection process, the benefit of the study, the confidentiality of their information, and their voluntary status for participating were given to the participants. Appending their signature signifies voluntary participation; this was obtained from all participants. Two research assistants, comprising final-year students of the Department of Physiotherapy, were recruited and tutored on the procedures of this research. They assisted in the distribution of the paper-based questionnaires (Health and Safety Work-related stress questionnaires, the Coping mechanism questionnaire, Work-Related Quality of life questionnaires, and general information form) with the informed consent form as the cover page to different departments, the retrieval of the questionnaires, and the collation of the completed questionnaire. An estimated period of four weeks was utilized to administer the questionnaires to the participants.

### Data analysis

The data were analyzed using IBM Statistical Packages for the Social Sciences (SPSS 23.0: SPSS Inc., Chicago, IL, USA). Data from this study were summarized using descriptive statistics of frequency count, percentages, mean, and standard deviation. The inferential statistics of Mann-Whitney and Kruskal Wallis tests were applied to analyze the difference in WRS, coping mechanisms, and WRQoL, by sex, academic rank, and years of teaching experience at an alpha level of 0.05.

## Results

A total of 283 participants were recruited for this study, consisting of 119 (42%) males. About 104 (37%) of the participants were divorced and the majority were ≥ 31 years (59.2%) of age with 45.8% having more than 10 years of teaching experience (Table 1). The mean score of work-related stress (WRS) and quality of work-life scores among the participants are 117.55 ± 10.53 and 3.94 ± 0.19 respectively as well as the mean score of coping mechanism subset (Self distraction, Denial, Use of emotional support, Use of instrumental support, Venting Planning Humor, Acceptance, Religion, Active coping, Substance use, Behavioral disengagement, Positive retraining, and Self blame) were shown in Table 2.

**Table 1:** Socio-demographic profile of the participants

Variables		Frequency	Percentage
Sex n = 283	Male	119	42.0
	Female	164	58.0
Marital status n = 282	Single	44	15.7
	Married	63	22.4
	Divorced	104	37.0
	Widowed	70	24.9
Age (Years ) n = 282	≤ 24	1	0.4
	25–30	114	40.4
	≥ 31	167	59.2
Teaching experience n = 276	2–5 years	48	17.4
	6–10 years	86	31.2
	11–15 years	69	25.0
	≥ 16 years	58	21.0
	≥ 25years	15	5.4
Faculty n = 283	Bioscience	17	6.0
	FHST	22	7.8
	Medicine	17	6.0
	Engineering	70	24.7
	EHS	38	13.4
	Agriculture	30	10.6
	Arts	20	7.1
	Basic medicine	18	6.4
	Law	4	1.4
	Education	14	4.9
	Management Sciences	5	1.8
	Pharmaceutical sciences	5	1.8
	Physical Sciences	7	2.5
	Social sciences	13	4.6
Academic rank n = 263	Professor	1	4
	Associate professor	7	2.5
	Senior lecturer	19	6.7
	Lecturer 1	100	35.3
	Lecturer 11	72	25.4
	Assistant lecturer	55	19.4
	Graduate assistant	9	3.2

**Table 2:** Mann-Whitney U test comparing coping, stress and quality of work life scores between male and female respondents

Variables	Mean ± Standard deviation	Mean rank (sex)		U	P
		Male	Female		
Coping					
Self distraction	5.39 ± 1.44	124.55	154.66	7681.50	0.002
Active coping	5.53 ± 1.42	128.34	151.91	8133.00	0.014
Denial	3.69 ± 1.26	132.82	148.66	8665.00	0.094
Substance use	2.48 ± 1.28	169.79	121.84	6451.00	0.000
Use of emotional support	5.25 ± 1.25	125.56	153.93	7802.00	0.003
Use of instrumental support	5.06 ± 1.33	123.91	155.13	7605.00	0.001
Behavioral disengagement	3.08 ± 1.35	165.56	124.90	6954.00	0.000
Venting	5.43 ± 1.62	128.09	152.09	8103.00	0.012
Positive retraining	5.69 ± 1.51	157.68	130.62	7892.00	0.005
Planning	4.90 ± 1.16	146.87	138.46	9178.00	0.374
Humor	3.38 ± 1.73	143.06	141.23	9632.00	0.849
Acceptance	5.10 ± 1.24	106.71	167.60	5559.00	0.000
Religion	4.46 ± 1.54	128.15	152.05	8110.00	0.011
Self blame	4.80 ± 1.35	155.35	132.06	8169.50	0.017
Work-related stress	117.55 ± 10.53	140.96	142.76	963,450	0.855
Quality of Work Life	3.94 ± 0.19	148.81	137.06	8947.50	0.231

Table 2 depicted that there was a significant difference in the coping subset (Self distraction, Active coping, Substance use, Use of emotional support, Use of instrumental support, Behavioral disengagement, Venting, Positive Difference retraining, Acceptance, Religion, and Self blame) between sex (p < 0.05). More female (Mean rank = 16.60) uses acceptance as a coping mechanism and more males use substance abuse (Mean rank = 169.79) as means of coping with stress. All coping mechanism subsets were statistically significant for differences in years of teaching experience and academic rank (P < 0.05) as shown in Tables 3 and 4.

**Table 3:** Mann-Whitney U test comparing coping, stress and quality of work life scores by Years of Teaching Experience

Variables	Mean rank (Years of Teaching Experience)					K	P
	2–5	6–10	11–15	16–24	>=25		
Coping							
Self distraction	110.96	155.52	135.54	147.25	108.87	13.39	0.010
Active coping	160.84	130.92	159.59	116.81	97.30	18.59	0.001
Denial	102.52	187.66	111.98	128.38	132.90	54.90	0.000
Substance use	114.00	144.20	116.14	183.25	114.00	67.87	0.000
Use of emotional support	135.77	140.80	145.22	140.23	96.43	5.173	0.003
Use of instrumental support	72.69	161.98	176.12	122.34	103.93	64.06	0.000
Behavioral disengagement	80.58	160.70	141.82	155.09	117.13	40.24	0.000
Venting	109.17	182.88	117.42	141.58	63.00	54.08	0.000
Positive retraining	132.59	114.06	103.48	202.61	210.73	75.38	0.000
Planning	63.05	192.13	115.64	142.66	161.50	96.21	0.000
Humor	71.89	150.38	152.51	171.74	90.60	55.75	0.000
Acceptance	93.88	169.55	159.11	130.51	39.40	62.19	0.000
Religion	59.81	165.96	164.25	143.40	95.47	74.77	0.000
Self blame	72.46	121.67	162.82	199.36	99.13	84.24	0.000
Work-related stress	127.81	139.27	133.66	148.24	152.90	4.72	0.000
Quality of Work Life	126.42	150.10	139.53	126.49	152.37	2.49	0.646

**Table 4:** Mann-Whitney U test comparing coping, stress and quality of work life scores by Academic Rank

Variables	Mean rank (Academic Rank)							K	P
	Professor	Associate Professor	Senior Lecturer	Lecturer I	Lecturer II	Assistant Lecturer	Graduate Assistant		
Coping									
Self distraction	235.50	171.00	166.92	131.77	111.64	144.75	102.00	17.38	0.008
Active coping	257.50	162.00	145.11	113.75	147.34	149.35	41.06	30.29	0.000
Denial	257.50	165.50	86.50	122.02	129.92	180.81	22.50	55.91	0.000
Substance use	104.00	104.00	192.37	126.52	136.24	124.23	104.00	30.52	0.000
Use of emotional support	48.50	48.50	127.50	140.40	97.57	160.05	226.50	51.04	0.000
Use of instrumental support	235.50	157.50	96.76	137.84	99.53	157.40	238.11	46.58	0.000
Behavioral disengagement	149.00	149.00	146.32	129.60	133.83	136.71	70.00	8.21	0.001
Venting	237.00	53.00	137.42	131.28	115.04	181.58	11.00	61.98	0.000
Positive retraining	151.00	151.00	244.05	146.79	83.49	124.05	151.00	80.37	0.000
Planning	243.00	147.50	104.21	130.64	130.23	154.26	59.50	19.54	0.003
Humor	19.50	62.57	172.50	130.02	121.02	146.97	131.33	18.00	0.006
Acceptance	133.50	5.00	106.50	137.86	122.60	149.35	188.50	33.97	0.000
Religion	46.50	123.00	164.05	118.82	138.47	122.06	236.28	27.91	0.000
Self blame	140.50	30.00	199.42	161.53	90.15	121.25	140.50	68.87	0.000
Work-related stress	68.00	168.29	141.13	123.9	137.21	141.48	81.28	8.94	0.177
Quality of Work Life	255.50	128.50	137.00	139.70	119.52	132.64	120.89	5.93	0.431

## Discussion

The main purpose of this study was to determine work-related stress, coping strategies, and work-related quality of life among lecturers in a tertiary educational institution in Nigeria and how sex, years of teaching experience, and academic rank of lecturers affects the aforementioned variables. The findings were discussed thus:

### Difference between male and female coping mechanisms, work-related stress, and quality of work life

The difference between male and female participants’ coping strategies was found to be statistically significant. The current study found that male lecturers were more tolerant of the working demands in contrast to their female counterparts. This could be subject to the dual role occupied by the female lecturers who combine their lecturing job and their role as mothers. This study revealed that the sex of lecturers affected their ability to adjust and adapt to their work demands. This finding was supported by the report of a previous study that male lecturers used less social coping than females and there is a significant difference in the coping to stress between male and female lecturers [25]. Also, our finding is in alignment with work done by Graves et al. [26] and Kalu et al. [27] that agreed on a difference in coping strategies between males and females in the workplace. Although some other works [28, 29] are in disagreement with this finding, we buttress this result on the basis that females are more emotion-focused than males. This can be seen with more females having acceptance as a coping mechanism and males using substance abuse. Although both males and females use all forms of coping strategy, the males seem to apply problem-focused and maladaptive coping strategies; while females use more emotion-focused and adaptive strategies.

There was no statistically significant difference between male and female participants’ work-related stress. This suggests that sex will not affect work-related stress scores meaning that all participants would experience the same work-related stress scores based on sex. Explicitly, being a male or a female doesn’t contribute to the level of work-related stress felt by the lecturers. However, a study stated that there was a weak relationship between work-related stress and gender [30]. Our finding agrees with two other studies which assert that sex does not have an influence on the stress level of an individual [28, 31], and other studies attributed their outcome to the fact that both the male and female lecturers experience the same kind of organizational pressure [32, 33]. Although the mean difference in sex is insignificant, females had a higher mean rank than their male counterparts. This result suggests that females are more prone to emotional instability than males. Calvarese [34] opined that more females encountered greater degrees of sorrow, disappointment, and nervousness when reacting to stress in comparison to their male counterparts. Our finding could also be due to the fact that more females participated in this study.

There was no statistically significant difference between male and female participants’ quality of work life. This implies that sex does not have any effect on the work-related quality of life of lecturers. This is contrary to the previous finding which stated that the quality of life of female teachers is worse than that of male teachers [35] and another study where they found a significant difference in the quality of life of their male and female subjects [36]. A study concluded that the risk assessment of the quality of working life should take into due account the individual peculiarities of workers, with an apt focus on sex [37]. We believe that this might depend on the environment of study, and the condition of service which could vary from one educational institution to the other. Where the environment of work and the condition of service are favorable to either the male or female lecturers, the quality of life could tilt.

### Difference in coping mechanisms, work-related stress, and quality of work life based on years of teaching experience

There was a statistically significant difference in coping mechanisms and years of teaching experience among the respondents. This implies that the lecturers’ years of practice do affect their coping experience. We tend to agree with this finding because we believe that the ability of an individual to adopt a favorable coping strategy might rely on experiences gathered over the years, thus aiding in acquainting one on what to expect on the job and reducing elements of unforeseen, which contributes to stress and coping. This is in agreement with a previous finding that lecturers who have practiced for 15 years and above have adopted adequate coping strategies over the years to deal with stress [25]. Lecturers who are more advanced in age tend to develop means of coping with the realities of the job in contrast to younger lecturers, and with increased years of practice grow the ability to cope with the presenting workload, job satisfaction, financial remunerations, and family issues [25]. We also speculate that because the promotion of lecturers in Nigeria’s educational system draws a greater percentage of points from the literary output, like the publication of research in scientific journals, younger lecturers are also laden with the issue of article publications to earn promotion, hence whittling their coping capacity.

The difference in work-related stress by years of teaching experience was found to be statistically significant. This posits that working experience has an effect on work-related stress. Those who have long working experience may feel more stress than those with lesser working experience and vice versa. The young lecturers who have not spent so many years in service might feel less work-related stress if the environmental factors that contribute to stress were controlled. Interestingly, this agrees with the finding of previous studies that reported differences in the stress outcome of lecturers who have spent long years and those who have spent lesser years [38–42]. Also, the finding of another study reported that the level of stress among experienced and inexperienced lecturers was significant because the stress was less experienced by those who have practiced for a longer time as they have adopted adequate coping strategies over the years to deal with the stress [25]. Therefore, it may be assumed that older people because of their work experience may have gotten more experience and a better strategy to cope with the intricacies of doing their daily routine.

The difference in the quality of work life by years of teaching experience was found not to be statistically significant. The implication is that the number of years spent lecturing does not influence the quality of life of the lecturers. This is in agreement with two previous studies that found no statistically significant effect of experience on QoWL [43, 44]. This could be a result of poor job satisfaction, an unmanageable student population, the poor interpersonal relationship among staff, and a lack of instructional resources. We speculate that the welfare package available to the lecturers can contribute significantly to their subsisting quality of life. If the welfare package is poor, it might impact the capacity of the respondents to have access to the good things in life that would contribute to their having an improved quality of life. This entails that being long in service is not a guarantee that lecturers have a good quality of life. However, previous studies have revealed that younger academics experienced more stress than their older counterparts [41, 42]. The reason for this may be due to the fact that quality of life is multifaceted and the year a person spent on a job may not necessarily translate to a better quality of work life and that other factors may be at play which was not the focus of this present study. Although the lecturers who have stayed longer on the job may have adopted adequate coping strategies to deal with stress and may also think that they are at the pinnacle of their career and so do not stress themselves beyond their limits, thus contributing to their quality of work life, this too did not affect our finding.

### Difference in coping mechanisms, work-related stress, and quality of work life based on academic rank

The difference in coping strategies by academic ranks was found to be statistically significant. This can be expected as increased years of practice come with high ranks. The high-ranking lecturers such as professors and senior lecturers may have understood the rigors of academics and so have a higher coping mechanism than the lower-ranking lecturers. We speculate that this finding may be true because the young lecturers who have not spent so many years in service might have a less coping strategy as their workloads are less. This disagrees with the finding of the previous study that reported no difference in the coping strategy of lecturers who have spent long years and those who have spent lesser years [42].

The difference among academic ranks’ work-related stress was found to not be statistically significant. This finding contrasted with a previous study that posits academic rank as a factor that may have an influence on work-related stress [42, 45]. The findings of other previous research showed that workers at lower organizational levels reported feeling more alienated than those working at higher levels, and they also reported experiencing less job satisfaction and more occupational stress [46–48]. A study found that handling crisis situations look more stressful for employees at higher organizational levels, when compared to workers at lower levels. Another finding was that employee who perceived they received poor remuneration and progression lag in their career is more stressed than employees who perceived that they got adequate salaries and careers. Although we did not find a statistically significant difference, our result still buttressed the importance of organizational rank as the main influence on the occupational stress experienced by men and women in different works of life and underpins the relevance of assessing the effects of organizational ranks on specific sources of work-related stress.

The difference among academic ranks’ quality of work life was found to not be statistically significant. This contrasted with the finding of a previous study that found that teachers’ QoWL was significantly different based on their designation on all dimensions except for Job satisfaction and job security. However, it agrees with our finding that professors have a high mean rank [43]. This result is also consistent with the findings in the literature revealing a significant relationship between the quality of work life and career/promotion [49]. Another study agrees with the finding of this present research asserting that there is no statistically significant effect academic rank has on QoWL and the researcher believes that the employees have similar awareness of QoWL, regardless of their demographic factors (gender, age, academic qualification, experience) [44].

## Conclusion

There was a statistically significant influence of sex, years of teaching experience, and academic rank on coping strategies of lecturers. Also, a statistically significant influence of years of teaching experience on work related stress of lecturers was ascertained and revealed that male lecturers coped better with the rigorous demands of the job in contrast to female lecturers.

## Limitation of study

The cross-sectional research design has several limitations coupled with a non-probability sampling strategy (Consecutive sampling) which prevents generalizability, although a proportionate stratified randomized sampling was used to select the proportion of lecturers from 101 departments. Also, the fact that the study was conducted only in one university affected the sample size. Also, the researchers were not in the position to verify the authenticity of the information provided by the respondents since the study was questionnaire-based hence; the outcome of the study should be interpreted with caution.

## Contribution to knowledge

We are of the opinion that the differences between the current study and previous studies with regards to how lecturers cope with their stressors might be informed by varying demographic background and incentives from the employers. The study revealed that lecturers were exposed to many stressors which could affect their work performance. The outcome highlighted different coping strategies adopted by the lecturers, especially the older ones to mitigate the effect of work-related stress. It seems that one of the strategies adopted by the senior and older lecturers as coping strategies was transferring some statutory academic responsibilities to the younger ones. This we speculate would burden the junior lecturers by increasing their level of work-related stress. Their coping strategies are influenced by sex, years of teaching experience, and academic rank. Contrary to the findings of some previous studies, the sex of the respondents did not determine the quality of work life and work related stress of the lecturers. The stressors may be mitigated if other factors determining the quality of work life of the lecturers can be correctly determined and handled satisfactorily. The lecturers should be thought coping strategies, especially the females to counter the effect of work-related stress so that they can give their best in tutoring the students and their overall well-being. Factors determining the coping mechanism among lecturers in NAU should be extensively studied to enable the environment to be conducive for lecturers to operate without hindrance. A randomized controlled trial involving lecturers in multi centers in Nigeria should be conducted in future studies to compare the current outcome.

## Data Availability

The data is with the corresponding author and will be made available on a reasonable request.

## Abbreviations

NAU: Nnamdi Azikiwe University
HSE-WRS: Health and Safety Work-related Stress
WRQL: Work-related Quality of Life
QoWL: Quality of Work Life
BCQ: Brief-cope Questionnaire
CM: Brief Cope
SPSS: Statistical Package for Social Sciences
